# The Role of Reactive Oxygen Species in Diabetes-Induced Anomalies in Embryos of Cohen Diabetic Rats

**DOI:** 10.1080/15604280214933

**Published:** 2002

**Authors:** Sarah W. Zangen, Pirhiya Yaffe, Svetlana Shechtman, David H. Zangen, Asher Ornoy

**Affiliations:** 1 Laboratory of Teratology Department of Anatomy and Cell Biology Hebrew University–Hadassah Medical School Jerusalem 91120 Israel; 2 Department of Pediatrics Hadassah University Hospital Jerusalem Israel

## Abstract

The role of the antioxidant defense mechanism in diabetesinduced anomalies was studied in the Cohen diabetes-sensitive (CDs) and -resistant (CDr) rats, a genetic model of nutritionally induced type 2 diabetes mellitus. Embryos, 12.5-day-old, of CDs and CDr rats fed regular diet (RD) or a diabetogenic high-sucrose diet (HSD) were monitored for growth retardation and congenital anomalies. Activity of superoxide dismutase (SOD) and catalaselike enzymes and levels of ascorbic acid (AA), uric acid (UA), and dehydroascorbic acid (DHAA) were measured in embryonic homogenates. When fed RD, CDs rats had a decreased rate of pregnancy, and an increased embryonic resorption. CDs embryos were smaller than CDr embryos; 46% were maldeveloped and 7% exhibited neural tube defects (NTDs). When fed HSD, rate of pregnancy was reduced, resorption rate was greatly increased (56%; *P* < .001), 47.6% of the embryos were retrieved without heart beats, and 27% exhibited NTD. In contrast, all the CDr embryos were normal when fed RD or HSD. Activity of SOD and catalase was not different in embryos of CDs and CDr rats fedRD. When fed HSD, levels of AA were significantly reduced, the ratio DHAA/AA was significantly increased, and SOD activity was not sufficiently increased when compared to embryos of CDr. The reduced fertility of the CDs rats, the growth retardation, and NTD seem to be genetically determined. Maternal hyperglycemia seems to result in environmentally induced embryonic oxidative stress, resulting in further embryonic damage.


*Int. Jnl. Experimental Diab. Res.*, 3:247–255, 2002
Copyright © 2002 Taylor & Francis
1560-4284/02 $12.00 + .00
DOI: 10.1080/15604280290013964

Taylor&Francis healthsciences

### The Role of Reactive Oxygen Species in Diabetes-Induced Anomalies in Embryos of Cohen Diabetic Rats

**Sarah W. Zangen,[1] Pirhiya Yaffe,[1] Svetlana Shechtman,[1] David H. Zangen,[2] and Asher Ornoy[1]**

[1]*Laboratory of Teratology, Department of Anatomy and Cell Biology, Hebrew University–Hadassah Medical School, Jerusalem, Israel*
[2]*Department of Pediatrics, Hadassah University Hospital, Jerusalem, Israel*

**The role of the antioxidant defense mechanism in diabetes-induced anomalies was studied in the Cohen diabetes-sensitive (CDs) and -resistant (CDr) rats, a genetic model of nutritionally induced type 2 diabetes mellitus. Embryos, 12.5-day-old, of CDs and CDr rats fed regular diet (RD) or a diabetogenic high-sucrose diet (HSD) were monitored for growth retardation and congenital anomalies. Activity of superoxide dismutase (SOD) and catalase-like enzymes and levels of ascorbic acid (AA), uric acid (UA), and dehydroascorbic acid (DHAA) were measured in embryonic homogenates. When fed RD, CDs rats had a decreased rate of pregnancy, and an increased embryonic resorption. CDs embryos were smaller than CDr embryos; 46% were maldeveloped and 7% exhibited neural tube defects (NTDs). When fed HSD, rate of pregnancy was reduced, resorption rate was greatly increased (56%; $P < .001$), 47.6% of the embryos were retrieved without heart beats, and 27% exhibited NTD. In contrast, all the CDr embryos were normal when fed RD or HSD. Activity of SOD and catalase was not different in embryos of CDs and CDr rats fed RD. When fed HSD, levels of AA were significantly reduced, the ratio DHAA/AA was significantly increased, and SOD activity was not sufficiently increased when compared to embryos of CDr. The reduced fertility of the CDs rats, the growth retardation, and NTD seem to be genetically determined. Maternal hyperglycemia seems to result in environmentally induced embryonic oxidative stress, resulting in further embryonic damage.**

**Keywords** Catalase; Congenital Anomalies; Embryos; Oxidative Stress; SOD; Type 2 Diabetes

The increased rate of fetal malformations in diabetic pregnancy despite better glycemic control represents a clinical problem and a research challenge. The exact mechanism behind the elevated rate of malformations is presently unknown. It has, however, been suggested that both intrauterine (maternal) environment as well as genetic background may be important [1–7] in the teratogenic process. As of today, the literature describes 3 main pathways by which diabetes may affect the normal development of the embryos: disturbed inositol uptake, yielding lowered intracellular inositol concentration [8]; diminished flow in the arachidonic acid–prostaglandin pathway, yielding decreased PGE2 concentration [9]; and excess amount of reactive oxygen species, resulting in decreased amount of low-molecular-weight antioxidants, such as vitamin C, uric acid, glutathione, and vitamin E [5, 6, 10, 11]. The reactive oxygen species pathway seems to be the most important pathway because antioxidants may protect against the disturbances of both the inositol and the arachidonic pathways. The proposed mechanisms for increased reactive oxygen species generation at higher glucose concentrations include nonenzymatic protein glycosylation [12–14], enhanced mitochondrial electron transport chain flow [15], autooxidation [16], and changes in the redox potential. The relative

Received 26 February 2002; accepted 22 July 2002.
This project was supported by grant no. 032-5196 from the Israel National Science Foundation; the AM Cohen Foundation for the advancement of research of Cohen diabetic rat. The authors acknowledge Dr. Bina Cohen and Mr. Moshe Cohen for continuing support and encouragement.
Address correspondence to Asher Ornoy, Laboratory of Teratology, Department of Anatomy and Cell Biology, Hebrew University–Hadassah Medical School, Jerusalem, Israel 91120. E-mail: Ornoy@cc.huji.ac.il immaturity of antioxidant defense mechanisms during early embryonic development [17] predisposes the embryo to heightened risk for oxidative attacks on proteins, DNA, and lipid membranes by reactive oxygen species [18].

The most important intracellular reactive oxygen species–scavenging enzymes are superoxide dismutase (SOD), which produces hydrogen peroxide from superoxide radicals, and catalase-related enzymes, which decompose hydrogen peroxide. In addition, there are lypophilic and hydrophilic low-molecular-weight antioxidants, such as vitamin C, uric acid, glutathione, and vitamin E, which act directly with various reactive oxygen species. Recent studies showed that the addition of SOD or antioxidants to "diabetic culture medium" reduces the rate of embryonic anomalies [11, 19–21]. Moreover, the activities of catalase-like enzymes [22] and SOD [10] were reduced in malformed embryos of diabetic rats. Our laboratory [17, 21] recently reported a decreased activity of SOD and catalase and a marked reduction in low-molecular-weight antioxidants in embryos from normal rats cultured in a "diabetic culture medium" containing high levels of glucose and ketone bodies.

This study aimed to investigate the antioxidant defense mechanism in the Cohen diabetic rat model [23, 24]. The Cohen diabetic rat model consists of 2 genetically derived contrasting strains: The Cohen diabetes-sensitive strain (CDs) and a diabetes-resistant strain (CDr). The CDs strain develops overt type 2 diabetes when fed a diabetogenic high-sucrose low-copper diet (HSD), but maintains normal blood glucose when fed regular rat diet (RD). In contrast, the CDr maintained normal glucose levels when fed HSD or RD [23, 24]. Previous in vitro studies demonstrated a higher rate of malformations and growth retardation in embryos of the hyperglycemic CDs rats than in embryos of the streptozotocin-induced diabetic rats when cultured under the same "diabetic environment" [27]. The authors suggested that embryopathy in the Cohen diabetic rat model was induced by a combination of genetic and environmental factors. Eriksson et al. also described a high rate of congenital malformations in a substrain of Sprague-Dawley rat, denoted U strain, that was developed spontaneously out of the H strain and did not develop congenital anomalies [7]. The catalase activity and levels of catalase and Mn-SOD mRNA were decreased in the U embryos when compared with that of the H embryos, reducing further when the mother was diabetic [5, 11, 22]. The authors suggested that the impaired expression of scavenging enzymes in response to reactive oxygen species excess might be genetically determined.

In the present study, we investigated if there is a basic difference in the rate of pregnancies and pregnancy outcome of CDs fed RD or HSD in comparison to CDr rats. In addition, we investigated if there was differences in the levels of ascorbic acid (AA) and uric acid and in the activities of the scavenging enzymes SOD and catalase in embryos of CDs and CDr rats fed RD or HSD. Previous studies carried out on this strain [1, 2, 25] showed a high percentage of malformations in embryos of the hyperglycemic CDs rats between gestational days 11 and 13. Other studies from our [17, 26] and other [27, 28] laboratories showed a gradual increase in SOD and catalase activities with embryonic age, reaching peak levels during neonatal period. Based on these studies, we chose to study 12.5-day-old embryos of CDs and CDr rats. The use of the Cohen diabetic rat model provides the unique opportunity to isolate the genetic predisposition from the environmental deleterious factors of diabetes. This enables us to compare the embryos of the normoglycemic diabetic-prone CDs rats (when fed RD) to the embryos of the hyperglycemic CDs rats (when fed HSD) and to the embryos of the diabetes-resistant CDr (fed RD or HSD) in relation to pregnancy rate and outcome, levels of low-molecular-weight antioxidants, and activities of SOD and catalase.

### MATERIAL AND METHODS

### Animals

*Maintenance*

Animals were housed 5 per cage and separated by gender, except for mating intervals. During pregnancy, females were housed in individual cages. Twelve-hour diurnal light-darkness cycles were maintained. Room temperature was kept between 22°C and 25°C. These conditions are in accordance with "principles of laboratory animal care" (National Institutes of Health [NIH] publication no. 85-23, revised 1985) and the guidelines of the American Society of Physiology for the care of laboratory animals. Each pregnant female in the experiment was monitored for the number of pregnancies, date of delivery, the percent of resorbed embryos (gestational sacs with only deciduas, but no embryos), and the percent of live embryos (embryos with a beating heart when scored under the dissecting microscope).

*Feeding Protocols*

Animals used in our study were separated from their mothers at 5 to 6 weeks of age, after which they were fed either "regular" rat chow or a "diabetogenic" high-sucrose diet, according to the study protocol. RD consisted of a mixture of ground whole wheat, ground alfalfa, bran, skimmed milk powder, and salts, resulting in 21% protein, 60% carbohydrates, 5% fat, and 0.45% NaCl content (Koffolk). Chow and tap water were provided ad libitum. HSD consisted of 18% casein, 72% sucrose, 4.5% butter, 0.5% corn oil, 5% salt (no. II USP), water, and a vitamin mixture. The diabetogenic diet was copper-poor (1.2 ppm), a requirement for CDs to develop the full diabetic phenotype [23, 24]. Chow and distilled water for drinking were provided ad libitum. Diabetes was confirmed in the CDs rats after 2 months on HSD, as serum glucose level was 300 to 400 mg/dL in the oral glucose tolerance test. The diabetic rats were not treated with insulin at any time during the experiment.

*Mating Protocol*

Females CDs and CDr rats, 14- to 16-week-old, fed HSD or RD, were mated overnight for 10 to 12 hours with males of the same strain. Males were always fed RD and thus both CDs and CDr males kept normal blood glucose levels. Vaginal smears were performed daily in a group of rats to determine periodicity of the estrus cycle. The day on which sperm were found in the vaginal smears was considered gestational day 0.

### Analysis of Morphological Changes

The pregnant rats were euthenized on gestational day 12.5. Embryos were quickly dissected from the uterus and were evaluated under a dissecting microscope for congenital anomalies and scored according to the method described by Brown and Fabro [29]. This included examination for heart beats, yolk sac circulation, axial rotation, presence of telencephalic hemispheres, optic and otic vesicles, body size, number of somites and brachial bars, neural tube closure, and other gross anomalies. The embryos were then removed and prepared for further analysis. For each of the study groups, embryos were grouped according to the following criteria: (1) "normal" embryos: embryos having fully rotated tails, with closed neural tube, and the stage of development of major organs appropriate for the gestational age; (2) NTD embryos: embryos having open neural tube; (3) gross anomalies: embryos having malrotation of their tails and/or one (or more) of their major organs absent or maldeveloped for their appropriate gestational age; and (4) maldeveloped embryos: embryos developmentally delayed for their specific gestational age.

### Preparation of Embryonic Samples

The embryos were processed individually, homogenized in 0.6 mL phosphate-buffered saline (PBS, pH 7.4) solution. The homogenized samples were then centrifuged at 10,000 $\times$ *g* for 5 minutes at 4°C; the supernatant was stored at −80°C, until further used. For SOD, catalase, AA, and uric acid measurements, embryos of animals fed HSD were divided into embryos with pulse and without pulse and data were compared within groups.

### Protein Measurements

Protein content was measured in the crude homogenate according to Bradford [30], using human serum albumin as standard.

### Detection of Antioxidant Enzyme Activities

*SOD Activity*

SOD activity was studied in embryonic homogenates by the methods described by McCord and Friedovich [31] and by Grankvist and colleagues [32]. Xanthine oxidase (Sigma, St. Louis, MO), 1 U/mL, added to the solution produced reactive oxygen species. Cytochrome *c* (Sigma), 3 mM, was used as a detector. The superoxide radicals reduced cytochrome *c*, and the reduced cytochrome *c* was measured by spectrophotometer (Uvicon 933, Kontron, Switzerland) at 550 nm. The addition of SOD reduced the amount of superoxide radicals, thereby reducing the amount of reduced cytochrome *c* detected. SOD activity was calculated from the slope obtained when optical density (OD) was plotted versus time. Enzymatic activity was studied for 2 minutes (the time for the reaction) and was expressed in units per minute per milligram protein. The incubation solution was PBS. The assay was conducted at 25°C without preincubation. Because we measured the activity in the entire embryonic homogenate, we could not differentiate between the activities of CuZn-SOD and Mn-SOD. Therefore, this method measured mainly CuZn-SOD, but also some of Mn-SOD, activity, and the data will be referred as total SOD activity.

*Catalase-Like Activity*

The method used was described by Thurman and colleagues [33]. Aliquots of 100 $\mu$L of the homogenate were added to the reaction mixture containing hydrogen peroxide at a concentration of 150 $\mu$M. The reaction mixture also contained 1 mM 3-amino-1,2,4-triazole (Sigma) to stop glutathione peroxidase activity. After 10 minutes of incubation at room temperature, the reaction was stopped by the addition of 200 $\mu$L 30% trichloroacetic acid (TCA), and the remaining hydrogen peroxide was determined according to the Thurman procedure. In brief, it measures the red complex formed by hydrogen peroxide, ferrous ammonium sulfate (Sigma), and 25% thiocyanate (Sigma). The concentration of the complex is read by a spectrophotometer at 480 nm, which is directly related to the concentration of hydrogen peroxide in the tested solution. A calibration curve of catalase was prepared separately, and the results were calculated as "catalase-like" activity. This method measures the activity of catalase along with other scavenging enzymes having a catalase-like activity, such as glutathione peroxidase.

### Measurement of Uric Acid and Ascorbic Acid

The content of uric acid and AA was assessed only in embryonic homogenates of CDs and CDr rats fed HSD. The content of uric acid, dehydroascorbic acid (DHAA), total ascorbic acid (after reduction by cysteine) were assessed using an high-performance liquid chromatography (HPLC) instrument equipped with an electrochemical detector [11, 21]. We also calculated the change in the ratio of DHAA over total AA (DHAA/totalAA), because DHAA is the oxidation product of ascorbic acid, and may therefore indicate oxidative stress.

### Statistical Analysis

Statistical analysis was performed using Student's *t* test (2-tailed) and analysis of variance (ANOVA) or their respective nonparametric tests when appropriate. Data were compared by Bonferroni *t* test. Significance was set at $P < .05$. ANOVA for one dependent variable by one or more factors and or variables was performed to study the effect of covariate interactions with the factors and their effect on the mean.

### RESULTS

### Pregnancy Profile of CDs and CDr Rats Fed RD or HSD (data not shown in Table 1)

Pregnancy rate and menstrual cycles were monitored in CDs and CDr females. Although both CDs and CDr rats, regardless of feeding protocol (RD or HSD), have normal menstrual cycles (each cycle lasting ∼5 days), CDs rats were less fertile than CDr rats when fed RD or HSD.

When rats were fed RD, successful mating (presence of sperm in the vaginal smears) was achieved in CDs females only after 2 to 6 mating trials and pregnancy was not achieved in 8% of CDs females that had sperm in the vaginal smears. Female CDs had less than 4 pregnancies per year and had an average of 6 live progenies per pregnancy. When fed HSD, only 4 out of the 26 females included in the study group became pregnant, which was achieved after an average of 9 mating trials. About 30% of the CDs females fed HSD that had sperm in the vaginal smears were not pregnant during the mating period of the study.

In contrast, CDr females achieved successful mating after 1 to 2 mating trials when fed RD, resulting in an average of 7 successful pregnancies. Female CDr had an average of 5 pregnancies per year and of 8 live progenies per pregnancy. Successful mating was achieved in CDr females fed HSD after 3 to 4 mating trials, resulting in 5 successful pregnancies. Only 1 of the 16 CDr females that had sperm in the vaginal smear was not pregnant.

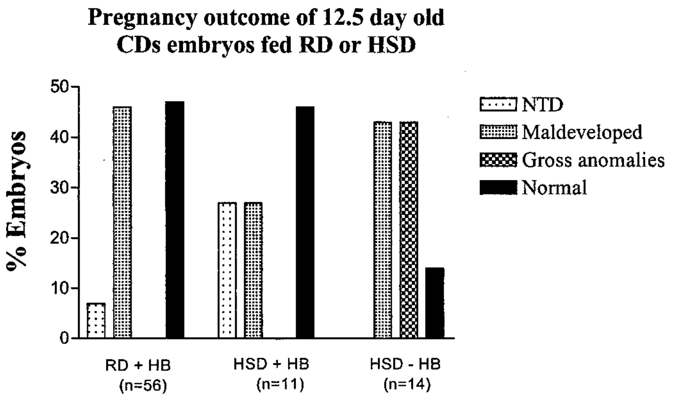

**FIGURE 1**
Pregnancy outcome of embryos of CDs rats fed RD or HSD. Embryos of CDs rats fed HSD were further divided to embryos with heart beats (+ HB) and embryos without heart beats (− HB). In each group, the percent (%) of embryos that were normal, maldeveloped, or had gross anomalies or NTD was assessed.

### Pregnancy Outcome of CDs and CDr Rats Fed RD or HSD

When fed RD, pregnancy rate of CDs rats was already decreased (28%) and was further significantly reduced to 15% ($P < .03$ versus CDs RD) when fed HSD (Table 1). Pregnancy rate of CDr rats was 47% ($P < .05$ versus CDs RD) when fed RD and decreased to 33% when fed HSD. A high percentage of resorbed embryos (24%) was found in CDs rats fed RD. Resorption rate was further increased to 56% ($P < .03$ versus CDs RD) when fed HSD. When fed RD, all (100%) the embryos of CDs rats were retrieved with heart beats (HB) but only 52.4% when fed HSD. In contrast, CDr rats maintained a low resorption rate (3%) when fed RD or HSD, all (100%) the embryos were retrieved with HB when fed RD, and most of them (82%) when fed HSD (Table 1). Seven percent of embryos of CDs rats exhibited neural tube defects (NTD) when fed RD, 46% were maldeveloped, and only 47% were not affected (Figure 1). When fed HSD, the percentage of NTD was tripled (27%) in embryos of CDs rats with HB, 27% were maldeveloped, and the remaining 46% had a lower score than embryos of CDs rats fed RD but were otherwise normal (Figure 1). In contrast, most (82%)

**TABLE 1**
Pregnancy outcome of embryos of rats fed RD or HSD

| | CDr RD | CDr HSD | CDs RD | CDs HSD |
|---|---|---|---|---|
| Number of females | 15 | 15 | 25 | 26 |
| Number of pregnancies | 7 (47%) | 5 (33)% | 7 (28%) | 4 (15%)$^{a,b}$ |
| Number of live embryos (% total) | 58 (100%)$^{a}$ | 23 (82%) | 53 (100%) | 11 (52.4%) |
| % resorption (total/implantation sites) | 3% | 3% | 24%$^{a,b}$ | 56%$^{a,b}$ |

$^{a}P < .03$ versus CDs RD; $^{b}P < .03$ versus CDr HSD.

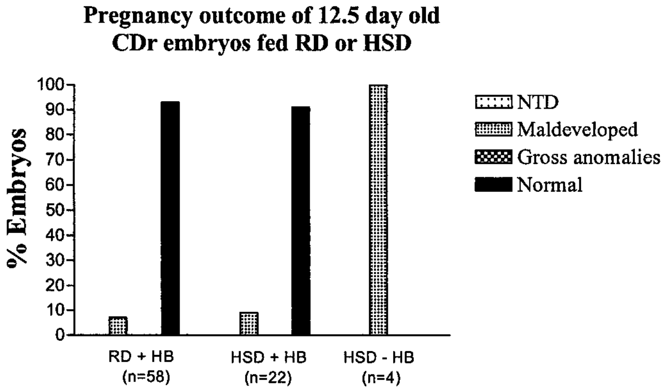

**FIGURE 2**
Pregnancy outcome of embryos of CDr rats fed RD or HSD. Embryos of CDr rats fed HSD were further divided to embryos with heart beats (+ HB) and embryos without heart beats (− HB). In each group, the percent (%) of embryos that were normal, maldeveloped, or had gross anomalies or NTD was assessed. Embryos of CDr rats did not exhibit any NTD or gross anomalies.

of the embryos of CDr rats fed RD or HSD were normal. Only 7% were maldeveloped when fed RD and 9% when fed HSD (Figure 2).

### Size (Crown-Rump Length [CRL]) and Average Score of Embryos With Pulse of Rats Fed RD or HSD (Table 2)

When animals were fed RD, embryos of CDs rats had a significantly smaller CRL ($P < .03$) and a lower score ($P < .001$) when compared to embryos of CDr rats (Table 2). When fed HSD, embryos of CDs rats with HB were significantly smaller ($P < .001$) and had a lower score ($P < .03$) when compared to embryos with HB of CDr rats fed HSD, or to embryos of CDs rats fed RD (Table 2). Moreover, the CRL of embryos of CDr rats increased significantly when the rats were fed HSD, whereas the CRL of embryos of CDs rat was not significant different from the CRL of embryos of CDs fed RD (Table 2).

**TABLE 2**
Crown-rump length (CRL) and score of embryos of rats fed RD or HSD with HB

| | Number of embryos | CRL (mm) | Score |
|---|---|---|---|
| CDs RD | 50 | $5.3 \pm 0.07$ | $43.0 \pm 0.17$ |
| CDs HSD (with HB) | 11 | $4.9 \pm 0.4$ | $39.4 \pm 1.7^{a}$ |
| CDr RD | 50 | $6.0 \pm 0.05^{a}$ | $44.3 \pm 0.09^{a}$ |
| CDr HSD (with HB) | 21 | $7.1 \pm 0.2^{b,c}$ | $53.2 \pm 1.2^{c}$ |

*Note*. CRL and score data are mean ± SE.
$^{a}P < .05$ versus CDs RD; $^{b}P < .05$ versus CDr RD; $^{c}P < .05$ versus CDs HSD with HB. HB = heart beats.

### Embryos With HB of Rats Fed RD or HSD

*Protein Content* (Table 3)

Protein content of embryos of CDs rats fed RD was significantly lower ($P = .038$) than that of embryos of CDr rats fed RD. Moreover, embryonic protein content was 3 times higher in embryos (with HB) of CDs rats fed HSD ($P < .03$), and 6 times higher ($P = .000$) in embryos with HB of CDr rats fed HSD, when compared with their respective strain fed RD.

*Activity of SOD and Catalase* (Tables 3, 4)

*Total SOD Activity* (Table 3). When fed RD, activity of SOD per embryo (unit/embryo/min) and (unit/mg protein/min) of the embryos of CDs rats was not significantly different than that of embryos of CDr rats fed RD. When fed HSD, activity of SOD increased significantly ($P = .03$) in both CDs and CDr rats' embryos with HB, when compared with their respective strain fed RD. When calculated as unit/mg protein/min, SOD activity was significantly reduced in embryos of CDr rats ($P = .03$), but was not significantly different in embryos of CDs rats. The significant reduction in SOD activity calculated per mg protein in the embryos of CDr rats may be due to the significant increase (6 times) in embryonic protein content.

*Catalase Activity* (Table 4). When fed RD, the activity of catalase per embryo (unit/embryo) was not significantly different in the CDs embryos when compared to embryos of CDr rats (Table 4). However, activity of catalase calculated as unit/mg protein was significantly higher ($P < .05$) in embryos of CDs rats when compared with embryos of CDr rats. The apparent increase in catalase activity in embryos of CDs rats may be due to the significant difference in embryonic protein between the strains (the reduction was in the embryos of CDs rats).

*Levels of Uric Acid, AA, and DHAA*

Uric acid content of embryos with HB of CDs rats fed HSD was not statistically different than that of embryos with HB of CDr rats fed HSD (Figure 3). Levels of AA were significantly reduced in embryos with pulse of CDs rats ($P < .001$) when compared with embryos of CDr rats. However, the ratio DHAA/totalAA of embryos with pulse of CDs rats were significantly higher than the ratio DHAA/totalAA of embryos with pulse of CDr rats ($P < .02$) (Figure 4).

### Embryos Without HB of Rats Fed HSD

Embryos of CDs without HB were significantly affected: 43% had major anomalies such as malrotation of the tail or absence of otic vesicle, 43% were maldeveloped, and only 14% had a smaller score than embryos of CDs rats fed RD but were otherwise normal (Figure 1). In contrast, only 4 out of the 27 embryos of CDr fed HSD were retrieved without HB. These

**TABLE 3**
Protein content and SOD activity of embryos with HB of rats fed RD or HSD

| | Number of embryos | Protein (mg/embryo) | SOD (unit/embryo/min) | SOD (unit/mg protein/min) |
|---|---|---|---|---|
| CDs RD | 50 | $0.20 \pm 0.02$ | $3.8 \pm 0.3$ | $13.3 \pm 1.8$ |
| CDs HSD (with HB) | 11 | $0.65 \pm 0.12^{a}$ | $6.7 \pm 1.5^{a}$ | $8.1 \pm 3.4$ |
| CDr RD | 51 | $0.27 \pm 0.03^{a}$ | $4.4 \pm 0.3$ | $14.7 \pm 2.1$ |
| CDr HSD (with HB) | 22 | $1.60 \pm 0.14^{a,b,c}$ | $8.6 \pm 1.1^{a,b,c}$ | $4.2 \pm 1.0^{a,b}$ |

*Note*. Protein content and SOD activity date are mean ± SE. SOD activity is expressed as both unit/embryo/min and unit/mg protein/min.
$^{a}P < .05$ versus CDs RD; $^{b}P < .05$ versus CDr RD; $^{c}P < .05$ versus CDs HSD with HB. HB = heart beats.

embryos had a lower score but did not exhibit any NTD or other malformations (Figure 2).

*Size (CRL) and Average Score* (Table 5)

Embryos without HB of CDs rats were significantly smaller ($P < .001$) than CDr rats embryos without HB, but their scores were not significantly different.

*Protein Content* (Table 6)

Embryonic protein levels did not increase in embryos without HB of CDr rats and was significantly reduced in embryos without HB of CDs rats ($P = .000$). Embryonic protein levels were also significantly reduced in these embryos when compared with embryos of CDs rats fed RD and to embryos with HB of CDs rats fed HSD ($P = .000$).

*SOD Activity* (Table 6)

When fed HSD, activity of SOD per embryo (unit/embryo/min) was significantly higher in embryos of CDr rats when compared with embryos without HB of CDs rats, but was not significantly different when calculated per milligram embryonic protein (unit/mg protein/min). SOD activity (unit/embryo/min) increased significantly ($P = .03$) in embryos without HB of CDr rats when compared with embryos of CDr rats fed RD, but was not different from the activity of embryos with HB of CDr rats fed HSD. No such increase was found in embryos without HB of CDs rats or when SOD activity was calculated per milligram embryonic protein.

**TABLE 4**
Catalase activity of embryos of rats fed RD

| | Number of embryos | Catalase (unit/embryo) | Catalase (unit/mg protein) |
|---|---|---|---|
| CDs RD | 49 | $5.9 \pm 0.2$ | $24.2 \pm 2.6$ |
| CDr RD | 49 | $5.8 \pm 0.3$ | $16.4 \pm 1.71$ |

Catalase activity is expressed as both unit/embryo and unit/mg protein and data are mean ± SE.
$^{a}P < .05$ versus CDs RD.

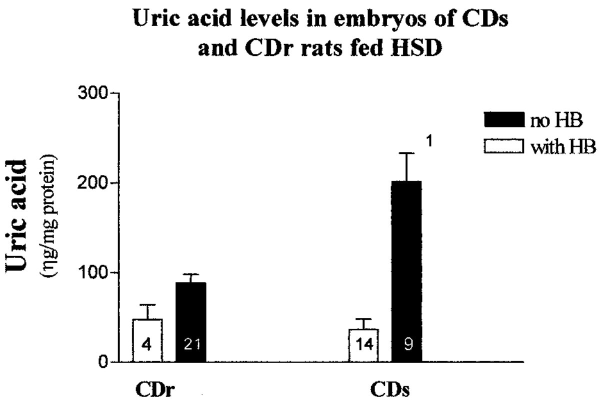

**FIGURE 3**
Levels of uric acid in embryos of CDs and CDr rats fed HSD. Levels of uric acid were compared between CDs and CDr rats and between embryos with heart beats (with HB) and without heart beats (no HB). $^{1}P < .05$ versus CDs with HB.

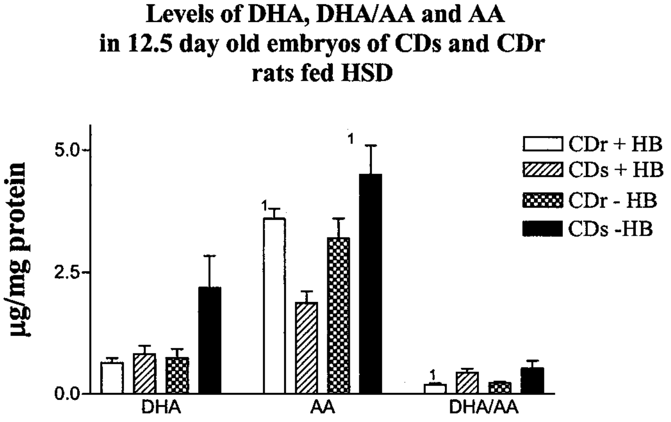

**FIGURE 4**
Levels of the low-molecular-weight antioxidants, dehydroascorbic acid (DHA) and ascorbic acid (AA), and the ratio DHA/AA in embryos of CDs and CDr rats fed HSD. Levels of low-molecular-weight antioxidants were compared between CDs and CDr rats and between embryos with heart beats (+ HB) and without heart beats (− HB). $^{1}P < .05$ versus CDs + HB.

**TABLE 5**
Crown-rump length (CRL) and score of embryos of rats fed HSD without HB

| | Number of embryos | CRL (mm) | Score |
|---|---|---|---|
| CDs HSD (no HB) | 10 | $2.9 \pm 0.3$ | $33.5 \pm 3.5$ |
| CDr HSD (no HB) | 4 | $5.9 \pm 0.1^a$ | $36.2 \pm 0.8$ |

*Note*. CRL and score data are mean ± SE.
[a] $P < .05$ versus CDs without HB. HB = heart beats.

*Levels of Uric Acid, AA, and DHAA* (Figure 3)

Uric acid content increased significantly in embryos without HB of CDs rats ($P = .021$) but not in embryos without HB of CDr rats (Figure 3) when compared with embryos with HB of CDs or CDr rats. Levels of AA of embryos without HB of CDs rats were not significantly different from AA levels of embryos with or without HB of CDr rats, but were significantly higher ($P < .02$) when compared with embryos with HB of CDs rats (Figure 4). The DHAA levels and the ratio DHAA/totalAA of embryos without HB of CDs rats were not significantly different from the levels of embryos with HB of CDs rats and from embryos with or without HB of CDr rats (Figure 4).

### DISCUSSION

In this study, we tried to differentiate between the genetic component—susceptibility to develop type 2 diabetes—and the effects of hyperglycemia per se on the embryo, by using the Cohen diabetic rat model. We showed that there was already a basic decrease in pregnancy rate and an increase in resorption rate in CDs rats when compared with CDr rats when rats were fed RD. Thus, the reduced fertility and the high rate of congenital malformations in the CDs rats seem to be genetically determined and preceed hyperglycemia. However, pregnancy rate of the normoglycemic CDs rats was still high enough to ensure normal continuity of the strain. The HSD, rendering the CDs rats diabetic, had no effect on the menstrual cycle but reduced pregnancy rate by half, doubled resorption rate, and affected pregnancy outcome. In contrast, pregnancy rate and outcome of CDr rats were almost not affected by HSD. A decrease in pregnancy rate has been shown in both human and experimental diabetes [2, 3, 6, 34] and was attributed to reduce fertility of both males and females. The reduced fertility observed in our study was mainly due to the females because the males were always maintained on RD and thus were not hyperglycemic. Moreover, the same pool of males that were used for mating the females on RD were used for mating the females fed HSD. We also showed that the embryos of CDs rats fed RD had a basic genetically determined retardation in growth and development when compared with embryos of CDr rats and exhibited 7% of NTD. Still, 93% of the embryos of normoglycemic CDs rats were viable (were retrieved with HB) and did not exhibit major anomalies. Previous studies performed on this strain describe a high rate of resorption, malformations, and growth retardation in embryos of CDs rats fed RD, which becomes more prominent in embryos of rats fed HSD during days 9 to 13 of gestation, but later in pregnancy some growth-retarded embryos recovered [2, 35]. This may indicate that during the later phases of gestation, the normal (but developmentally retarded) embryos of CDs rats, as observed in the high rate in our study, may have the ability to recover and catch up. These previous studies set the foundation for our present study, except that in previous studies, embryos of CDs rats were compared with embryos of Hebrew University control rats and to embryos of diabetic streptozotocin-induced rats. In the present study, we compared the outcome of pregnancies of the prediabetic CDs rats with that of the overt diabetic CDs rats and with that of the diabetes-resistant CDr rat.

The basic SOD activity, calculated either per embryo (unit/embryo/min) or per mg protein (unit/mg protein/min), was not significantly different in embryos of CDs rats fed RD when compared with embryos of CDr rats. These results are supported by the recent study of Cederberg and colleagues [5] showing that there was no difference in the basic level of the scavenging enzyme CuZn-SOD in embryos of the diabetic malformation-resistant strain (H) when compared with embryos of the diabetic malformation-prone strain (U).

Basic catalase activity was significantly increased in embryos of CDs rats only when calculated per mg protein. This may be explained by the fact that embryonic protein levels were significantly higher in the embryos of CDr rats and therefore

**TABLE 6**
Protein content and SOD activity of embryos of rats fed HSD without HB

| | Number of embryos | Protein (mg/embryo) | SOD (unit/embryo/min) | SOD (unit/mg protein/min) |
|---|---|---|---|---|
| CDs HSD (no HB) | 10 | $0.11 \pm 0.03^a$ | $4.4 \pm 1.6$ | $23.4 \pm 11.5$ |
| CDr HSD (no HB) | 5 | $0.4 \pm 0.08^b$ | $7.4 \pm 0.5^b$ | $11.5 \pm 2.6$ |

Protein content and SOD activity data are mean ± SE. SOD activity is expressed as both unit/embryo/min and unit/mg protein/min.
[a] $P < .05$ versus CDs RD; [b] $P < .05$ versus CDs without HB. HB = heart beats.

the activity per mg protein was apparently increased in CDs rats' embryos. Cederberg and colleagues [5] also described a basic difference in catalase activity between their H and U embryos. However, in their study, catalase activity per milligram embryonic protein was significantly higher in the H embryos. Cederberg and colleagues postulated that this basic difference in catalase activity might be due to different catalase isoenzymes in the 2 strains or to different amounts of enzyme protein per embryonic cell. Because protein levels were significantly decreased in CDs rats' embryos, we conclude that basic catalase activity was not different between strains. Therefore, we suggest that the basic scavenging enzyme activity in embryos of CDs and CDr rats fed RD was not significantly different.

HSD elicited a significant increase in embryonic protein content in both CDs and CDr rats' embryos with HB. However, this increase was significantly more prominent in CDr rats' embryos (a 3-fold increase in CDs rats' embryos versus a 6-fold increase in CDr rats' embryos). SOD activity increased significantly in embryos with HB of both CDs and CDr rats fed HSD, only when calculated per embryo but not when calculated per mg protein. A possible explanation is that the SOD activity we measured in the homogenates of the entire embryo represents the total activity of both CuZn-SOD and Mn-SOD. However, usually changes in Mn-SOD activity alone account for activity increases that accompany differentiation, whereas changes in CuZn-SOD activity occur postnatally [27]. Therefore, the increase in Mn-SOD, if present, may have been undetected when measured as total SOD activity. This explanation is supported by the studies of Cederberg and colleagues [5, 36] showing that the increased activity of SOD in embryos of the malformation-resistant strain, as a result of maternal diabetes, was mainly due to increase in Mn-SOD and not to CuZn-SOD.

We found that when rats were fed HSD, AA concentration was significantly reduced in embryos (with HB) of CDs rats when compared with embryos (with HB) of CDr rats. Moreover, the ratio DHHA/totalAA was significantly higher in embryos of CDs with HB. This may suggest an excess of reactive oxygen species production in embryos of the hyperglycemic CDs, ensuing an increase in the utilization of AA, and hence a decrease in its concentration. A similar decrease in the low-molecular-weight antioxidants was described in recent in vitro studies performed in our laboratory on 10.5-day-old embryos of normal Hebrew University rats cultured for 28 hours in a medium containing high concentration of glucose and ketone bodies ("diabetic medium" [11]). This study described a decreased concentration of water- and lipid-soluble low-molecular-weight antioxidants in these embryos that may indicate that the "diabetic medium" or serum reduced the antioxidant defense capacity of the embryos. The reduction in AA and the increase in DHHA/totalAA described in the present study may therefore suggest that the embryos of the hyperglycemic CDs rats are under oxidative stress. Therefore, because the CDs rats are developing overt hyperglycemia and oxidative stress when fed HSD, we anticipated a significant increase in SOD activity in their embryos to overcome the deleterious effects of hyperglycemia. However, SOD activity was either not increased (when calculated per protein) or not increased enough (when calculated per embryo) to protect the embryos from developing a high rate of NTD and maldevelopment. Furthermore, the activity of SOD was not different from that of embryos of the (normoglycemic) CDr rats fed HSD, and thus not increased enough in the live embryos (with HB) of CDs rats to protect them from developing malformations. Still, about 50% of the embryos of CDs rats fed HSD survived and 73% of the live embryos, although smaller and maldeveloped, did not exhibit anomalies. This may suggest an impaired SOD activity in embryos of the hyperglycemic CDs rats, which is clearly not the only factor responsible for the deleterious effect of hyperglycemia on the embryos of CDs rats. The HSD per se is also not solely responsible for the teratogenesis found in the CDs embryos, because it did not elicit any teratogenic effects in the embryos of CDr rats fed HSD. In the future, we plan to try to reduce the embryonic maldevelopment and anomalies by treating the hyperglycemic CDs rats with insulin to reduce the diabetic state and with the antioxidants vitamins C and E to reduce the level of embryonic oxidative stress.

Altogether, this study reconfirms in the genetically determined Cohen diabetic rats some of the findings in other animal models and adds new insight to the known teratogenicity of pregnancy of this strain. From the results of the present study, we may conclude that the decreased antioxidant defense capacity of embryos of the hyperglycemic CDs rats may explain only partially the deleterious effects of HSD on their pregnancy rate and outcome. However, to elucidate the precise nature of these conditions, studies, including expression of SOD and catalase mRNA and other regulatory transcriptional factors, are currently being carried out in our laboratory.

### REFERENCES

[1] Ornoy, A., Zusman, I., Cohen, A. M., and Shafrir, E. (1986) Effect of Sera from Cohen, genetically determined diabetic rats, sterptozotocin diabetic rats and sucrose fed rats on in vitro development of early somite embryos. *Diabetes Res.,* **3,** 43–51.

[2] Zusman, I., and Ornoy, A. (1986) The effect of maternal diabetes and high sucrose diets on the intrauterine development. *Diabetes Res.,* **3,** 153–159.

[3] Carrapato, M. R., and Marcellino, F. (2001) The infant of diabetic mother: The critical developmental windows. *Early Pregnancy,* **5,** 57–58.

[4] Schaefer-Graf, U. M., Buchanan, T. A., Xiang, A., Songester, G., Montoro, M., and Kjos, S. L. (2000) Patterns of congenital anomalies and relationship to initial glucose fasting levels in pregnancies complicated by type 2 diabetes. *Am. J. Obstet. Gynecol.,* **182,** 313–320.

[5] Cederberg, J., Galli, J., Luthmann, H., and Eriksson, U. J. (2000) Increased mRNA levels of Mn-SOD and catalase in embryos of diabetic rats from malformation resistant strain. *Diabetes,* **49,** 101–107.

[6] Eriksson, U. J., Borg, L. A., Cedesberg, J., Nordstrand, H., Siman, C. M., Wentzel, C., and Wentzel, P. (2000) Pathogenesis of diabetes induced congenital anomalies. *Ups. J. Med. Sci.,* **105,** 53–84.

[7] Eriksson, U. J. (1988) Importance of genetic predisposition and maternal environment for the occurence of congenital malformations in offspring of diabetic rats. *Teratology,* **37,** 365–374.

[8] Wentzel, P., Welsh, N., and Eriksson, U. J. (1999) Developmental damage, increased lipid peroxidation, diminished cyclooxygenase-2 gene expression, and lowered prostaglandin E2 levels in rat embryos exposed to a diabetic environment. *Diabetes,* **48,** 813–820.

[9] Wentzel, P., Wentzel, C. R., Gareskog, M. B., and Eriksson, U. J. (2001) Induction of embryonic dysmorphogenesis by high glucose concentration, disturbed inositol metabolism, and inhibited protein kinase C activity. *Teratology,* **63,** 193–201.

[10] Sivan, E., Reece, E. A., Wu, Y. K., Homko, C. J., Polansky, M., and Borenstein, M. (1997) Dietary vitamin E prophylaxis and diabetic embryopathy: Morphologic and biochemical analysis. *Am. J. Obstet. Gynecol.,* **175,** 793–799.

[11] Zaken, V., Kohen, R., and Ornoy, A. (2001) Vitamins C and E improve rat embryonic antioxidant defense mechanism in diabetic culture medium. *Teratology,* **64,** 33–44.

[12] Gillery, P., Monboisse, J. C., Maquart, F. X., and Borel, J. P. (1989) Does oxygen free radical increased formation explain long term complications of diabetes mellitus? *Med. Hypotheses,* **29,** 47–50.

[13] Hunt, J. V., Dean, R. T., and Wolff, S. P. (1988) Hydroxyl radical production and autoxidative glycosylation. Glucose autoxidation as the cause of protein damage in the experimental glycation model of diabetes mellitus and ageing. *Biochem. J.,* **256,** 205–212.

[14] Baynes, J. W. (1991) Role of oxidative stress in development of complications in diabetes. *Diabetes,* **40,** 405–412.

[15] Yang, X., Borg, L. A., and Eriksson, U. J. (1997) Altered metabolism and superoxide generation in neural tissue of rat embryos exposed to high glucose. *Am. J. Physiol.,* **272,** E173–E180.

[16] Wolff, S. P., and Dean, R. T. (1993) Diabetes mellitus and free radicals. Free radicals, transition metals and oxidative stress in the aetiology of diabetes mellitus and complications. *Br. Med. Bull.,* **49,** 642–652.

[17] Zaken, V., Kohen, R., and Ornoy, A. (2000) The development of antioxidant defense mechanism in young rat embryos in vivo and in vitro. *Early Pregnancy,* **4,** 110–123.

[18] Fantel, A. G., Barber, C. V., Carda, M. B., Tumbic, R. W., and Mackler, B. (1992) Studies of the role of ischemia/reperfusion and superoxide anion radical production in the teratogenicity of cocaine. *Teratology,* **46,** 293–300.

[19] Eriksson, U. J., and Borg, L. (1991) Protection by free oxygen radical scavenging enzymes against glucose-induced embryonic malformations in vitro. *Diabetologia,* **34,** 325–331.

[20] Eriksson, U. J., and Borg, L. (1993) Diabetes and embryonic malformations. Role of substrate-induced free oxygen radical production for dysmorphogenesis in cultured rat embryos. *Diabetes,* **42,** 411–419.

[21] Ornoy, A., Zaken, V., and Kohen, R. (1999) Role of reactive oxygen species (ROS) in the diabetes-induced anomalies in rat embryos in vitro: Reduction in antioxidant enzymes and low-molecular-weight antioxidants (LMWA) may be the causative factor for increased anomalies. *Teratology,* **60,** 376–386.

[22] Cederberg, J., and Eriksson, U. J. (1997) Decreased catalase activity in malformation-prone embryos of diabetic rats. *Teratology,* **56,** 350–357.

[23] Cohen, A. M. (1990) Development of the model. In: *The Cohen Diabetic Rat,* Edited by Cohen, A. M., and Rosenmann, E., pp. 1–9. Basel: Kargel.

[24] Weksler-Zangen, S., Yagil, C., Zangen, D. H., Ornoy, A., Jacob, H. J., and Yagil, Y. (2001) The newly inbred Cohen diabetic rat: A nonobese normolipidemic genetic model of diet-induced type 2 diabetes expressing sex differences. *Diabetes,* **50,** 2521–2529.

[25] Ornoy, A., Shechtman, S., Yaffe, P., and Weksler-Zangen, S. (2000) The role of reactive oxygen species in diabetes induced anomalies in embryos of the Cohen diabetic rat. Abstract 380. In: *Proceedings of the 36th Annual Meeting Of the European Association for the Study of Diabetes (EASD)*. Jerusalem, Israel.

[26] Ornoy, A., Kimyagarov, D., Yaffee, P., Abir, R., and Kohen, R. (1996) Role of reactive oxygen species in diabetes induced embryo-toxicity studies on pre-implantation mouse embryos cultured in serum from diabetic pregnant women. *Isr. J. Med. Sci.,* **6,** 634–639.

[27] Allen, R. G., and Balin, A. K. (1989) Oxidative influence on development and differentiation. An overview of a free radical theory of development. *Free Radic. Biol. Med.,* **6,** 631–639.

[28] Frank, I. (1991) Developmental aspects of experimental pulmonary oxygen toxicity. *Free Radic Biol. Med.,* **11,** 463–499.

[29] Brown, N. A., and Farbo, S. (1981) Quantitation of rat embryos development in in-vitro morphological scoring system. *Teratology,* **24,** 65–78.

[30] Bradford, M. (1976) A rapid and sensitive method for quantitation of microgram quantities of protein utilizing the principle of protein-dye binding. *Anal. Biochem.,* **72,** 248–254.

[31] McCord, J. M., and Friedovich, I. (1988) Superoxide dismutase: The first twenty years 1968–1988. *Free Radic. Biol. Med.,* **5,** 263–369.

[32] Grankvist, K., Marklund, S. L., and Täljedal, I. B. (1981) Cu, Zn-superoxide dismutase, catalase and glutathione peroxidase in pancreatic islets and other tissues in the mouse. *Biochem J.,* **199,** 393–398.

[33] Thurman, R. G., Ley, G. G., and Scholtz, R. (1972) Hepatic microsomal ethanol oxidation. Hydrogen peroxidase formation and the role of catalase. *Eur. J. Biochem.,* **25,** 420–430.

[34] Ornoy, A., and Cohen, A. M. (1980) Teratogenic effects of sucrose diet in diabetic and non diabetic rats. *Isr. J. Med. Sci.,* **16,** 789–791.

[35] Ornoy, A., and Zusmann, I. (1990) Embryonic development in the Cohen diabetic rats. In: *The Cohen Diabetic Rat,* Edited by Cohen, A. M., and Rosenmann, E., pp. 101–121. Basel: Karger.

[36] Forsberg, H., Borg, L. A., Cagliero, E., and Eriksson, U. J. (1996) Altered levels of scavenging enzymes in embryos subjected to a diabetic environment. *Free Radic. Res.,* **24,** 451–459.

